# Effect of NaCl on the luminescent behavior of CsI thin films

**DOI:** 10.1039/d5ra05682a

**Published:** 2025-10-06

**Authors:** Saurabh Singh, Xiyu Wen, Fuqian Yang

**Affiliations:** a Materials Program, Department of Chemical and Materials Engineering, University of Kentucky Lexington KY 40506 USA ssi303@uky.edu; b Center for Aluminium Technology, University of Kentucky Lexington KY 40506 USA

## Abstract

In recent years, several reports have shed light on the growing interest in modulating the optoelectronic properties of cesium iodide (CsI) thin films for enabling their application in low-cost and eco-friendly luminescent devices. However, traditional techniques used for such modification like annealing, doping, and nano-structuring, often demand high temperatures or vacuum processing, limiting their large-scale scalability. Herein, we report a simple, eco-friendly method to tailor the luminescent properties of CsI thin films by incorporating NaCl *via* a simple aqueous based, low-temperature (∼50 °C) process. CsI/NaCl films prepared in a 1 : 1 molar ratio of CsI and NaCl, exhibited strong UV-excited photoluminescence at ∼415 nm, with significantly longer radiative decay times (*τ*_3_ = 63.2 ns) than pure CsI films (*τ*_3_ = 27.94 ns), indicating suppressed non-radiative recombination likely due to partial defect passivation by Na^+^ ions at grain boundaries or surfaces. Moreover, a weaker broad emission, attributed to deep trap states, was also observed at ∼531 nm under this UV excitation of ∼365 nm wavelength. Notably, humidity-controlled studies revealed that both PL intensity and emission wavelength increase with relative humidity (RH) up to ∼50%, then decline at higher humidity due to moisture-induced defects and Na^+^ ion migration. Furthermore, XRD and SEM/EDS analyses confirmed mixed-phase domains and interface-rich regions that contributed to such moisture sensitive behavior of these films. Overall, this low-cost, solution-based strategy offers a scalable route to tune optical properties and stability in halide films *via* green technology for optoelectronic applications.

## Abstract 2

Schematic graphical abstract for CsI/NaCl (1 : 1) films depicting non-homogeneous distribution of CsI and NaCl across the films and their corresponding effect on photoluminescence behavior.

## Graphical abstract

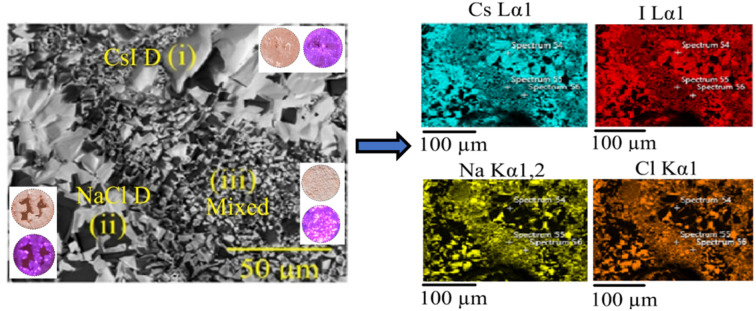

## 1. Introduction

Since the early 20th century, cesium iodide (CsI) has drawn enduring interest across fields such as scintillation and photodetection, owing to its high quantum efficiency, fast decay time, high atomic density, high light yield, efficient UV absorption, and robust stopping power.^1–7^ However, the broader application of CsI thin films is often constrained by their high hygroscopicity,^8^ making them prone to various challenges including increased sensitivity to humid environments,^9^ radiation-induced damages^10^ and poor film adhesion.^11^

Several efforts have been dedicated toward luminescent CsI thin films to address such challenges, such as Sheng *et al.*^12^ coated CsI thin films with an ultra-thin alumina (Al_2_O_3_) layer to protect them against their inherent hygroscopic nature while maintaining optical transparency. On the other hand, Farzaneh, *et al.*^11^ employed a sol gel technique to produce CsI thin films, demonstrating high quantum efficiency with homogeneous and uniform morphology, however, it was countered with some issues such as sensitivity to processing conditions^11^ and possibility of defect formation during shrinkage.^13^ Recently, Huang, *et al.*^14^ reported a simple green approach to produce blue emitting CsI thin films with a dominant photoluminescence (PL) peak at ∼415 nm along with a small peak at ∼536 nm depicting deep trap state emission, and they observed a decaying trend of PL intensity with increase in fabrication temperature, with the CsI films prepared at 20 °C depicting the most intense luminescence.

Several approaches have focused on modifying the opto-electronic properties of CsI,^1,2,6,15,16^ with the most common approach involving doping of rare earth or transition metal ions into CsI lattice to enhance its luminescent characteristics, including effective light yield and emission spectra, with common dopants including (Eu^+^),^16,17^ (Yb^2+^),^18^ (Tl^+^),^19,20^ and (Na^+^).^1,21,22^ For instance, Sofich, *et al.*^18^ successfully grew CsI crystals doped with Yb^2+^ through vacuum deposition and observed improved optical behavior of CsI-Yb^2+^ compared to pure CsI crystals in terms of higher luminescence yield and longer decay times. Moreover, Hsu, *et al.*^1^ reported a series of continuing work, focusing on the fabrication of CsI:Na thin films *via* thermal vacuum deposition followed by the deposition of protective layers (such as SiO_2_, Al, and parylene-N layers) for mitigation of moisture-induced degradation and correspondingly, they noticed a significant enhancement in luminescent behavior of CsI:Na thin films under both UV light and X-ray irradiation in comparison to pure CsI thin films. However, such doping approach *via* vacuum deposition often requires elevated temperatures, high cost, and long fabrication times, which limits their large scale scalability.^1,18^

Herein, we report a simple and cost-effective approach to investigate the influence of NaCl on the luminescent behavior of CsI thin films, fabricated *via* a green, solution-processed drop-casting method at ∼50 °C under varying humidity conditions. This study reveals the change in structural, morphological and opto-electronic behavior of CsI upon introduction of different amounts of NaCl in form of thin films. Moreover, controlled-humidity PL measurements revealed a crucial role of NaCl in governing the luminescent stability of CsI/NaCl films, with 50% relative humidity (RH) being identified as the optimal condition for the enhanced performance. Further experimentation and analysis demonstrated that NaCl incorporation significantly improves the optoelectronic properties of CsI thin films by suppressing non-radiative recombination and enhancing the photoluminescence efficiency.

## 2. Experimental details

### 2.1 Preparation of CsI/NaCl films

**Fig. 1:**
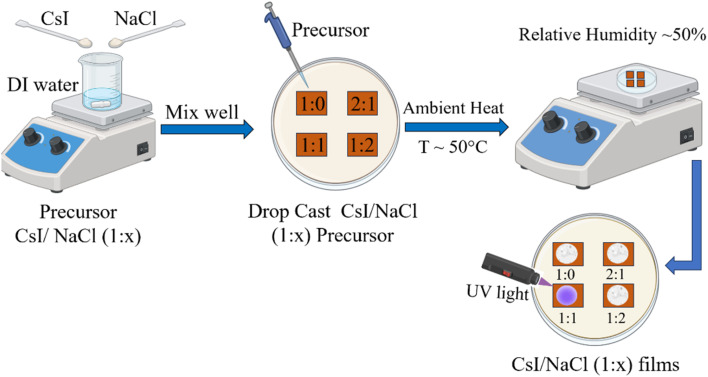
Schematic showing the preparation of a CsI/NaCl (1 : *x*) thin film.

Four different samples were prepared from aqueous solutions of CsI and NaCl with four different ratios (mole by mole) of CsI (purity: 99.9%, Alfa Aesar) to NaCl (99% dried, Macron Fine Chemicals) at a temperature of 50 °C. Specifically, 1 : 0, 1 : 1, 1 : 2, or 2 : 1 molar ratio was taken for CsI to NaCl powder; and the powders were then dissolved in 2 ml of DI water to form an aqueous solution. Finally, 100 μL of the prepared solution was dropped onto a copper substrate at 50 °C under ambient conditions under varying relative humidity levels (20–70%) and dried for ∼30 min to form a CsI/NaCl film. A schematic depicting the experimental process is shown in Fig. 1.

### 2.2 Materials characterization

The structural characterization of as-prepared CsI/NaCl films was performed on an X-ray diffractometer (XRD) (Siemens D500) with CuKα radiation (*λ* = 1.5406 Å). The morphological and chemical compositional studies were conducted on a Scanning Electron Microscope (SEM) (JEOL JSM-5900lLV) coupled with an energy-dispersive X-ray spectroscope (EDS). The X-ray Photoelectron spectroscopy (XPS) (Thermo Scientific K-alpha X-ray photoelectron spectrometer) was conducted to verify the chemical composition of the as-prepared CsI/NaCl films. The controlled humidity tests were performed using a vacuum desiccator (napco E series) and the PL spectra of the as-prepared CsI/NaCl films were obtained on a spectrometer (USB 2000+, Ocean Optics) with the UV excitation wavelength of 365 nm. Absolute PLQYs of samples were measured using Horiba Scientific Fluoromax Plus-C fluorometer. A solid sample was mounted on a sample stage and placed in a closed integrating sphere. An excitation wavelength of 365 nm was used for PLQY measurements with a 1 nm slit width and 0.1 s integration time. PLQY calculations were done using a Horiba Scientific FluorEssence™ software, respectively. The lifetime decay studies were conducted using a DeltaHub™ high throughput time correlated single photon counting (TCSPC) controller. Note that, for the PL studies, saturation region was considered intentionally for the measurements due to the detection limit of instrument and to have a clear experimental detail.

## 3. Results and discussion

**Fig. 2:**
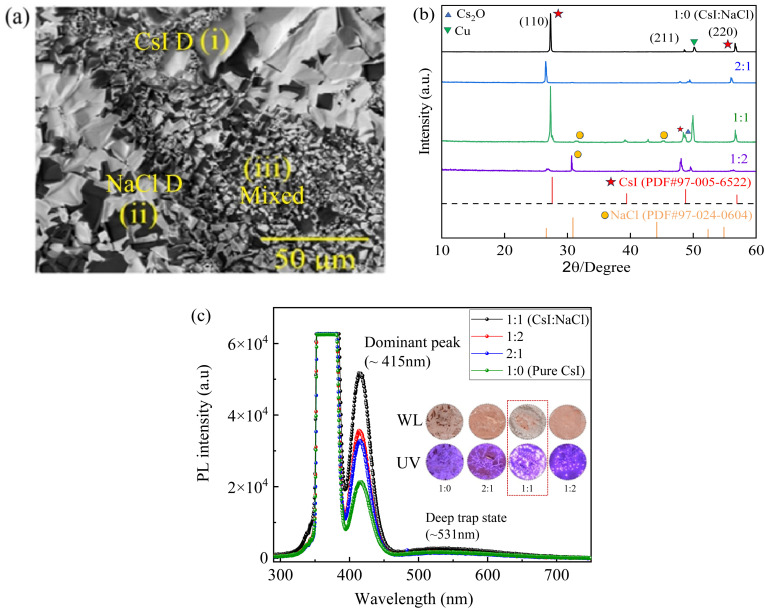
(a) SEM image of a CsI/NaCl film prepared from the aqueous solution with 1 : 1 molar ratio of CsI to NaCl at ∼50 °C and ∼50% optimized RH, depicting three regions: (i) large, bright CsI-rich grains; (ii) faceted dark NaCl grains; and (iii) fine-grained mixed-phase particles, (b) XRD spectra, (c) PL spectra with optical images as insets under white light (WL) and UV light (∼365 nm), for CsI/NaCl in 4 different molar ratios.

SEM (scanning electron microscopy) studies conducted on CsI/NaCl (1 : 1) films prepared *via* aqueous solution of CsI and NaCl in 1 : 1 molar ratio and deposited at 50 °C and an optimized RH of ∼50%, revealed three morphologically distinct regions: (i) large, bright CsI-rich grains; (ii) faceted dark NaCl grains; and (iii) fine-grained mixed-phase particles (Fig. 2a), as confirmed by EDS mapping (Fig. S1 and Tables S1–S3). These regions may arise from the dynamics of nucleation and growth during solvent evaporation at 50 °C and 50% RH, where, CsI, despite having a higher aqueous solubility than NaCl,^23,24^ likely reaches local supersaturation earlier, leading to its preferential nucleation and growth into larger grains, while, NaCl likely crystallizes later on from the residual solution forming well-faceted darker grains. In regions where nucleation events overlap or phase separation is incomplete, smaller mixed-phase particles form, likely featuring interfacial strain or structural disorder. Such spatial heterogeneity has been reported in multicomponent salt systems undergoing evaporation-driven crystallization.^25,26^

Fig. 2b shows the XRD spectra for the CsI/NaCl thin films prepared with the molar ratios of 1 : 2, 1 : 1, and 2 : 1 of CsI to NaCl under similar experimental conditions. For comparison, the XRD pattern of pure CsI film (1 : 0) prepared under the same conditions is also included in Fig. 2b. There are distinct peaks centred at ∼27.34°, 48.57°, 56.65° and 56.8° for the pure CsI film matching the cubic structure of CsI (PDF card #97-005-6522). It is interesting to note that all CsI/NaCl films exhibited a shift in CsI characteristics peaks to lower diffraction angles, indicating towards lattice expansion of CsI, and a shift in NaCl characteristic peaks to higher diffraction angles, suggesting lattice contraction of NaCl. However, the magnitude of shift in CsI peaks was found to be dependent on the amount of NaCl used during film preparation. Specifically, the CsI/NaCl (2 : 1 molar ratio) films depicted largest shift in CsI peaks to lower angles followed by CsI/NaCl (1 : 2 molar ratio) films, while CsI/NaCl (1 : 1 molar ratio) films exhibited the least shift in CsI peaks to lower angles.

These results suggest that NaCl interact structurally with CsI, likely through interfacial strain or limited incorporation of Na^+^ ions into the CsI lattice or defect sites, causing a distortion of host lattice leading to its corresponding expansion. Concurrently, the observed rightward shifts in NaCl peaks may arise from interfacial compressive strain or defects formation such as Na^+^ vacancies leading to lattice contraction due to inward relaxation of surrounding anions, thereby reducing average lattice spacing.^27,28^ These results were reproducible and reported well in SI (Fig. S3), confirming the structural consistency of these films across different batches.

It is worth noting that the peak (48.7°) on the right of the (211) peak (48.57°) of cubic CsI is assigned to Cs_2_O.^29^ During the drop-casting at 50°, Cs_2_O was formed. The formation of Cs_2_O can also cause the changes of the lattices of CsI and NaCl in the films. Moreover, the peak at ∼50° comes from Cu substrate used for making these thin films. Although, copper is known to form CuCl_2_ under certain environmental conditions, however, in our case, the combination of rapid drying at 50 °C and the presence of a passivating oxide layer on the copper surface prevented such a reaction.^30^ This is further experimentally supported by the absence of CuCl_2_ peaks in XRD studies (Fig. S4) and lack of any bluish-green region in optical images (Fig. S4) of pure NaCl film prepared by drop-casting an aqueous solution of NaCl onto a Cu substrate and drying under similar environmental conditions as of CsI/NaCl films.

Fig. 2c shows the PL spectra of the CsI/NaCl films under UV light of 365 nm in wavelength and it was evident that all the films emit blue light centred at ∼415 nm (∼2.99 eV) under ∼365 nm UV excitation. Among them, the CsI/NaCl films prepared *via* molar ratio of 1 : 1 (CsI : NaCl) exhibited highest PL intensity, suggesting an optimized balance between CsI and NaCl compositions. Moreover, EDS compositional mapping of the CsI/NaCl (1 : 1) films (Fig. S1 and Tables S1–S3), revealed three different regions in these CsI/NaCl (1 : 1) films, namely (i) bright CsI-rich grains; (ii) NaCl-rich grains; and (iii) fine-grained mixed-phase particles, suggesting non-uniform mixing of CsI and NaCl throughout these films. Furthermore, independent PL studies conducted on CsI/NaCl films prepared *via* EDS-derived compositions from regions (i) to (iii), revealed that the mixed-composition region (iii) (Fig. 2a and Table S3) exerted the most significant influence on the observed photoluminescence (PL) of CsI/NaCl films, directly correlating the incorporation of NaCl with the enhanced PL observed for these films, as shown in Fig. S2, respectively. Therefore, these analysis revealed that although CsI and NaCl are mixed together in a non-uniform manner forming separate CsI and NaCl domains (Fig. 2a, regions (i) and (ii)), they still exhibited enhanced PL due to presence of mixed-phase domains (Fig. 2a, region (iii)), and achieving this controlled and uniform distribution of both components (CsI and NaCl) across these films is inherently difficult and will be an important point of interest for our future studies.

Although the excitation energy (∼3.40 eV) is lower than the reported energy bandgap of bulk CsI (5.9–6.4 eV),^31^ however, similar sub-bandgap excitations have been effectively employed in prior studies on luminescent CsI thin films, where PL emissions in the 410–420 nm range were observed under 220–300 nm excitation sources.^11,32^ These sub-bandgap energy states emerge due to lattice strain, dislocations, halide vacancies, grain boundaries, and other microstructural defects formed during film growth^33,34^ particularly when prepared on metallic substrates like copper.^14^Fig. 2c (insets) depicts the optical images of as-synthesized CsI/NaCl films under white light and UV light (*λ* ∼ 365 nm), demonstrating the blue emission from all films under UV, regardless of the presence of NaCl, however, the intensity of emission changes significantly by varying the NaCl amount, respectively.

The excitation at 365 nm effectively promotes electrons to these defect-related or localized energy levels, enabling radiative recombination responsible for the observed PL at 415 nm which is consistent with previous reports attributing similar emissions in CsI films to microstructural defects and tensile lattice strain.^14,33,34^ For instance, Huang, *et al.*^14^ utilized 365 nm excitation was utilized for effective sub-bandgap excitations resulting in PL emission at ∼415 nm for pure CsI thin films fabricated using similar thermal evaporation method. There is also a small and broad PL peak at ∼531 nm for all the films which may be attributed to deep-level emission (DLE) arising likely due to self-trapped excitons or iodine vacancy related localized states and such a behaviour is well known characteristics of wide band gap halides such as CsI due to strong electron–phonon coupling.^14,35^ Moreover, all PL measurements were acquired under rigorously consistent conditions (identical excitation intensity, slit widths, integration times, *etc.*), allowing reliable relative luminescence comparisons across varying NaCl concentrations. The absolute photoluminescence quantum yield (PLQY) for these CsI/NaCl films turned out to be ∼44% at excitation wavelength of ∼365 nm (Fig. S5), which is significantly higher than CsI films deposited *via* thermal evaporation in high vacuum environments (3 × 10^7^ torr) by Rai, *et al.*,^36^ where author observed a decrease in absolute quantum efficiency (QY) of CsI films with increasing excitation energies, recorded in a UV spectral region of 150–200 nm, with maximum QE of ∼40% at an excitation wavelength of ∼150 nm, respectively. Moreover, further improvements can be made for quantum efficiency of these CsI/NaCl films *via* different surface passivation strategies such as polymer coating and encapsulation techniques, given the powder/rough nature of these films usually leads to stronger light scattering and relatively lower luminescence yields.^14,37,38^

Using Bragg's law^39^ for the XRD peak centred at ∼56.65° ((220) plane), we obtain the corresponding lattice constant of 4.63 Å, 4.74 Å, 4.64 Å, and 4.65 Å of the CsI/NaCl films from the aqueous solutions with *x* = 0, 0.5, 1, 2 for 1 : *x* of the molar ratio of CsI to NaCl and a comparative analysis of PL intensity with these lattice constants is presented in Fig. S6, respectively. Notably, the CsI/NaCl films prepared *via* 1 : 1 molar ratios, exhibited the least lattice strain and the largest PL peak intensity (Fig. S6), consistent with similar observation reported by Hsu, *et al.*,^1^ where the author demonstrated a change in optical behavior of CsI films with the strain introduced inside CsI lattice *via* external factors such as doping with Na^+^ ions in different amounts, and the films prepared using 1 : 1 (wt%) ratios of CsI and NaI exhibited least tensile strain in CsI lattice and the highest PL intensity compared to all other ratios. However, we note that such interpretations remain phenomenological and establishing a direct mechanistic link between induced strain and optical behaviour of these films would require in-depth theoretical analysis and simulations, which will be focus of future work. Overall, the subsequent studies in this work focus on the CsI/NaCl films with a 1 : 1 molar ratio deposited at 50 °C and ∼50% RH, respectively.

**Fig. 3:**
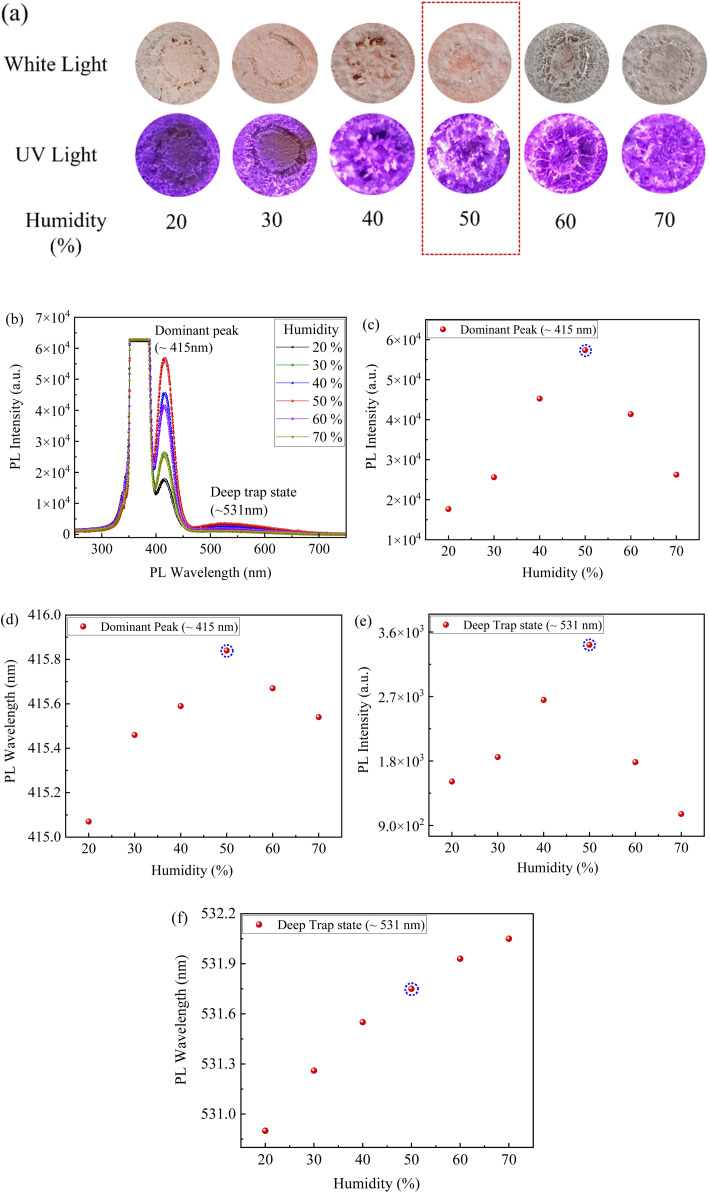
(a) Optical Images under white and UV light (∼365 nm), (b–f) PL spectral studies, under different humid environments, for CsI/NaCl films prepared *via* aqueous solutions of CsI to NaCl in 1 : 1 molar ratio.

Humidity-controlled photoluminescence (PL) measurements conducted on CsI/NaCl (1 : 1) thin films revealed a non-monotonic dependence on ambient moisture, as shown in Fig. 3a–d. Increasing relative humidity (RH) from 20% to 50% enhanced both PL intensity and dominant peak (∼415 nm) emission wavelength (Fig. 3a–d), consistent with trap passivation or Na^+^-mediated activation. This is consistent with XRD results of CsI/NaCl (1 : 1) films (Fig. 2b) deposited at 50% RH showing a left-shift in the CsI characteristic peaks compared to pure CsI films, indicating lattice expansion likely from partial Na^+^ incorporation or strain induced at CsI/NaCl grain boundaries. However, at higher RH (60–70%) levels, PL intensity tends to decline (Fig. 3c) and emission shifted back in wavelength (Fig. 3d), indicating moisture-induced luminescence quenching^40^ which is well consistent with previous reports on CsI:Na single crystals, where higher relative humidity (≥50–75% RH) promoted outward Na^+^ diffusion, forming Na-rich surface domains and leaving behind Na-deficient and optically inactive bulk regions and consequently, quenching the luminescence performance significantly.^40^

For instance, Yang, *et al.*^40^ reported the significant degradation in luminescence performance for CsI:Na crystals, as relative humidity increased from 50% to 75%, attributed to enhanced formation of optically inactive regions caused by outward migration of Na^+^ ions under elevated humidity conditions. Moreover, this decaying trend of PL intensity for CsI/NaCl (1 : 1) films (Fig. 3c) at higher RH levels (>50%) may attribute to potential hygroscopic and deliquescence characteristics of NaCl. As RH approaches the deliquescence threshold of NaCl (∼75% RH), this moisture uptake intensifies leading to partial dissolution of NaCl, Na^+^ ion migration, and chemical transformation at the film surface^40–42^ and such processes are known to increase the non-radiative recombination and phase separation, ultimately quenching the PL efficiency,^40–42^ as shown in Fig. 3a–c. Furthermore, a similar trend was observed for secondary deep-trap emission (∼534 nm), where maximum PL intensity was observed at ∼50% RH (Fig. 3e) and in contrast, it showed a continuous red-shift in wavelength with increasing RH (Fig. 3f). This suggests that lattice disorder and ion migration induced *via* humidity progressively activate the deeper trap states. At moderate humidity (20–50%), these deep traps may be partially passivated or stabilized, while at higher humidity (60–70%), enhanced defect formation and surface hydration likely deepen the trap states further, leading to red shift of PL wavelength (Fig. 3f) and an overall reduction in luminescent intensity (Fig. 3e).

**Fig. 4:**
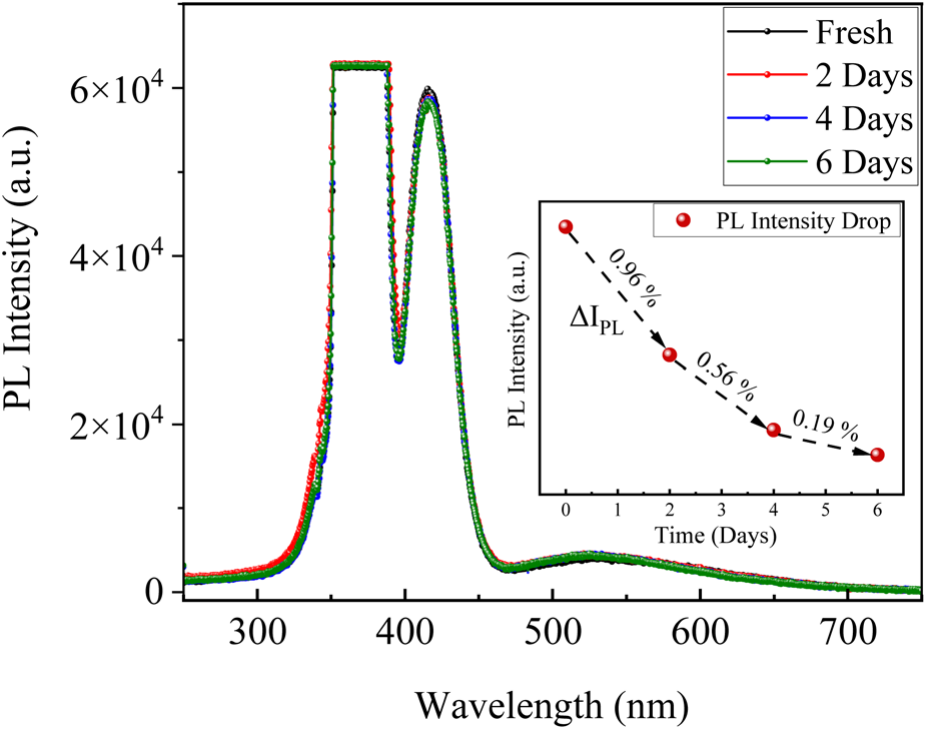
Photoluminescence (PL) Spectra of CsI/NaCl (1 : 1) films prepared at ∼50 °C and relative humidity of ∼50% under vacuum drying for period of 6 days (inset: temporal variation of relative PL Intensity (Δ*I*_PL_) for CsI/NaCl (1 : 1) films under vacuum drying).

Furthermore, to assess the effectiveness of drying at 50 °C, CsI/NaCl (1 : 1) films, prepared at ∼50 °C and relative humidity of ∼50%, were subsequently vacuum dried for 6 days and during which their PL intensity decreased marginally by ∼1.7% only, as shown in Fig. 4, at the end of 6th day respectively. This minimal PL change indicated that most moisture was already removed by the initial thermal drying step. This interpretation is well-supported by previous literature reports, which demonstrated that presence of even small moisture traces in hygroscopic CsI materials can lead to significant PL quenching, surface cracking, and degradation of luminescence performance of these materials.^1,43^ Therefore, the observed PL stability of CsI/NaCl (1 : 1) films, prepared at ∼50 °C and relative humidity of ∼50%, despite extended vacuum exposure, supports the conclusion that residual moisture does not significantly affect the reported optical behavior, affirming the adequacy of our drying protocol. Moreover, Huang, *et al.*^14^ recently reported excellent ambient stability of dried blue emitting CsI films prepared *via* similar drop casting method at room temperature and ambient conditions which indicated towards the absence of significant moisture inside such films, despite the known hygroscopic nature of CsI, further validating our interpretation.

**Fig. 5:**
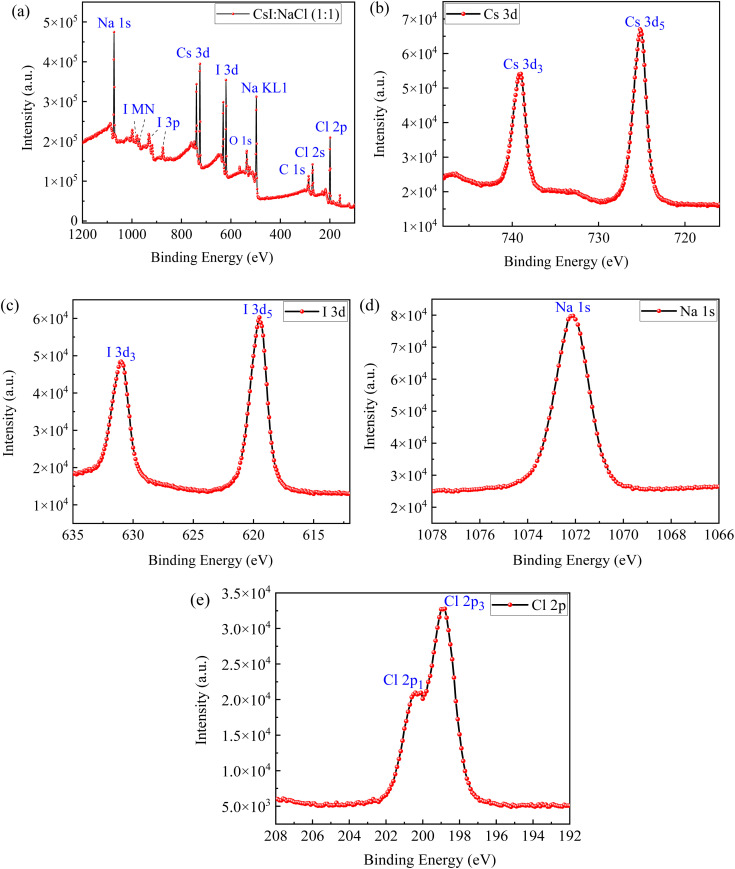
XPS survey spectrum of a CsI/NaCl film made from the aqueous solutions with 1 : 1 of CsI to NaCl with (a) combined XPS spectra, (b) elemental spectra for Cs 3d, (c) elemental spectra for I 3d, (d) elemental spectra for Na 1s, (e) elemental spectra for Cl 2p, respectively.

Fig. 5 presents the XPS survey spectrum of a CsI/NaCl film prepared *via* aqueous solutions with 1 : 1 molar ratio of CsI to NaCl, which includes the combined XPS survey spectrum (Fig. 5a) as well as high resolution spectra of individual elements, namely Cs 3d (Fig. 5b), I 3d (Fig. 5c), Na 1s (Fig. 5d) and Cl 2p (Fig. 5e). The characteristic peaks of Cs 3d, I 3d, Na 1s and Cl 2p are clearly visible confirming the presence of Cs, I, Na and Cl in these thin films (Fig. 5b–e). The double peaks observed in the high-resolution spectra of Cs 3d (Fig. 5b), I 3d (Fig. 5c), and Cl 2p (Fig. 5e) arises from spin–orbit splitting caused due to total angular momentum ‘*J*’. The presence of C and O (as indicated by C 1s and O 1s peaks) may attribute to outside system as well as the formation of Cs_2_O (Fig. 5b). Similar peaks were also observed in pure CsI thin films.^14^

A shift in the Cs 3d peaks towards lower binding energy was observed in CsI/NaCl films compared to pristine CsI and such binding energy shift may arise due to change in local chemical environment of Cs^+^ ions *via* interaction with NaCl, likely altering the coordination, interfacial interactions, or charge redistribution at the grain boundaries. This is consistent with literature where several studies have demonstrated that lattice strain and structural distortions can indirectly alter the local chemical environment of atoms and thereby affecting the effective charge density, Madelung potential, and relaxation energy and ultimately, influencing XPS peak positions.^44,45^ For instance, Richter, *et al.*^45^ demonstrated a direct relationship between lattice strain and core-level binding energy shifts arising due to alteration in the chemical bonding between metal atoms caused by strained lattice environments. Moreover, the presence of clear and distinct peaks of NaCl confirms the presence of these species in the CsI/NaCl (1 : 1) film. There is no major shift in I 3d peak positions pointing towards the preservation of ionic character of the species.

Time-resolved photoluminescence (TRPL) spectroscopy was employed to analyse the recombination kinetics of charge carriers in the prepared CsI/NaCl thin films. The temporal evolution of the TRPL intensity can be expressed as the sum of tri-exponential decay functions as:^46,47^

1 *I*_TRPL_(*t*) = *A*_1_ e^−*t*/*τ*_1_^+ *A*_2_ e^−*t*/*τ*_2_^ + *A*_3_ e^−*t*/*τ*_3_^

Here, *A*_*i*_ (*i* = 1, 2 and 3) and *τ*_*i*_ are the amplitude and lifetime of the *i*-th component, respectively.

**Fig. 6:**
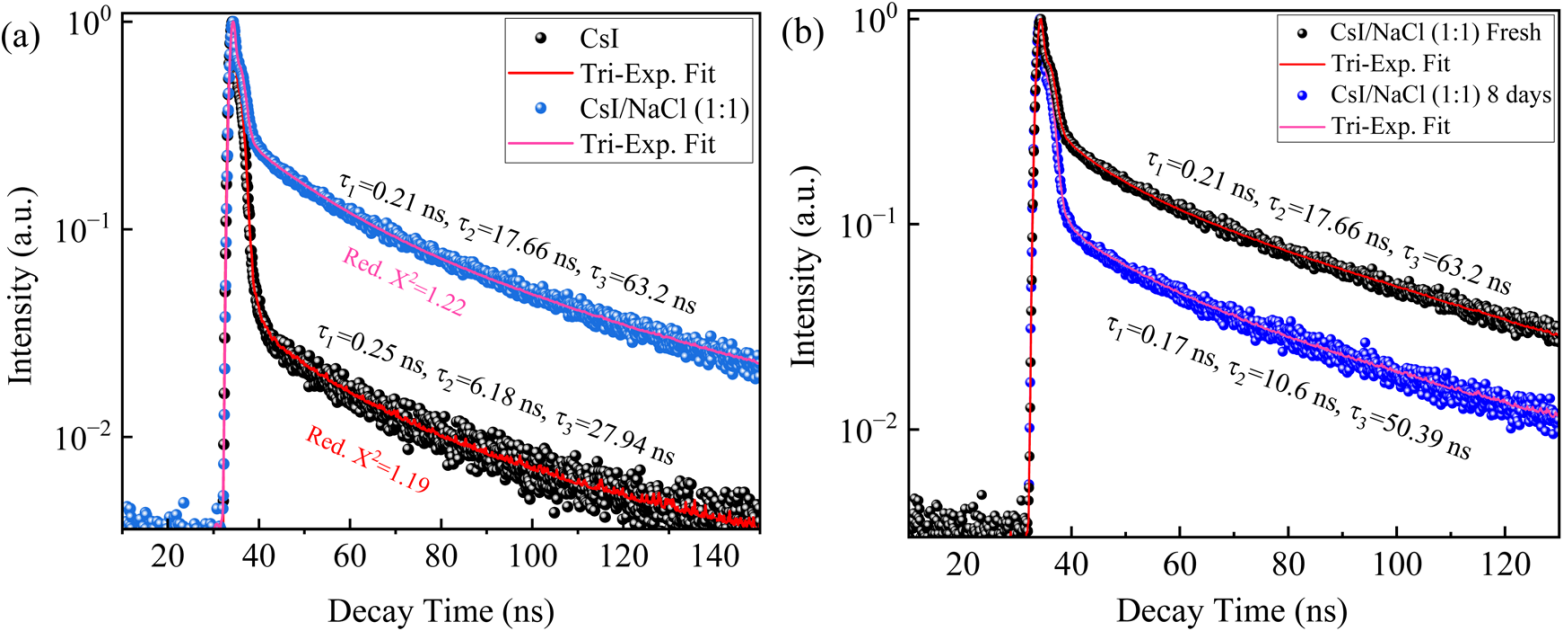
Time-resolved photoluminescence (TRPL) decay curves of (a) CsI and CsI/NaCl (1 : 1) films prepared at 50 °C and ∼50% relative humidity, and (b) CsI/NaCl (1 : 1) films measured fresh and after 8 days of ambient exposure (stored at ∼20 °C and ∼50% relative humidity).

Fig. 6a depicts the transient PL decay curves for pure CsI and CsI/NaCl (1 : 1) films and using eqn (1) to fit the TRPL decay curves, we obtain three lifetime values of 63.2 ns, 17.66 ns and 0.21 ns for the CsI/NaCl (1 : 1) thin film, corresponding to radiative, surface and trap-mediated recombination processes, respectively.^47^ The 63.2 ns for the radiative recombination for the CsI/NaCl (1 : 1) film is longer than 27.94 ns for pure CsI thin film suggesting a clear enhancement in charge carrier dynamics upon NaCl incorporation into CsI films. This trend implies a strong suppression of non-radiative pathways, likely due to passivation of intrinsic defects of CsI lattice by Na^+^ and Cl^−^ ions such as halide vacancies and interstitials and which are well known to act as non-radiative recombination centers by trapping charge carriers and ultimately quenching the PL performance.

This interpretation is consistent with XRD results (Fig. 2b), where leftward shift in CsI peaks were observed upon introduction of NaCl likely suggesting lattice distortion or ion incorporation, and enhanced PL intensity of CsI films upon NaCl incorporation (Fig. 2c), consistent with reduced trap-assisted recombination processes. Such behavior aligns well with past literature reports, such as Hsu, *et al.*^1^ demonstrated that Na doping in CsI films leads to enhanced luminescence and altered crystallinity by introducing new luminescent centers and reducing defect density, while, Zang, *et al.*^48^ showed that NaCl post-treatment of Sb_2_(S,Se)_3_ films significantly improved the PL intensity and increases the radiative decay lifetime by approximately 38%, which is attributed to effective defect passivation and improved film quality. Similarly, Abdi-Jalebi, *et al.*^49^ reported that potassium ion (K^+^) passivation in mixed-cation halide perovskites significantly enhanced the photoluminescence quantum efficiency (PQE) and suppresses the non-radiative recombination by passivating halide vacancies and mitigating trap-assisted recombination pathways. Notably, even after 8 days of ambient exposure, the CsI/NaCl (1 : 1) film maintained a radiative lifetime of 50.39 ns (Fig. 6b), indicating not only improved initial radiative recombination but also long-term stability which points towards partial retention of defect passivation even after 8 days, and which is significantly higher than fresh CsI films (27.94 ns), as shown in Fig. 6a and b, respectively.

**Fig. 7:**
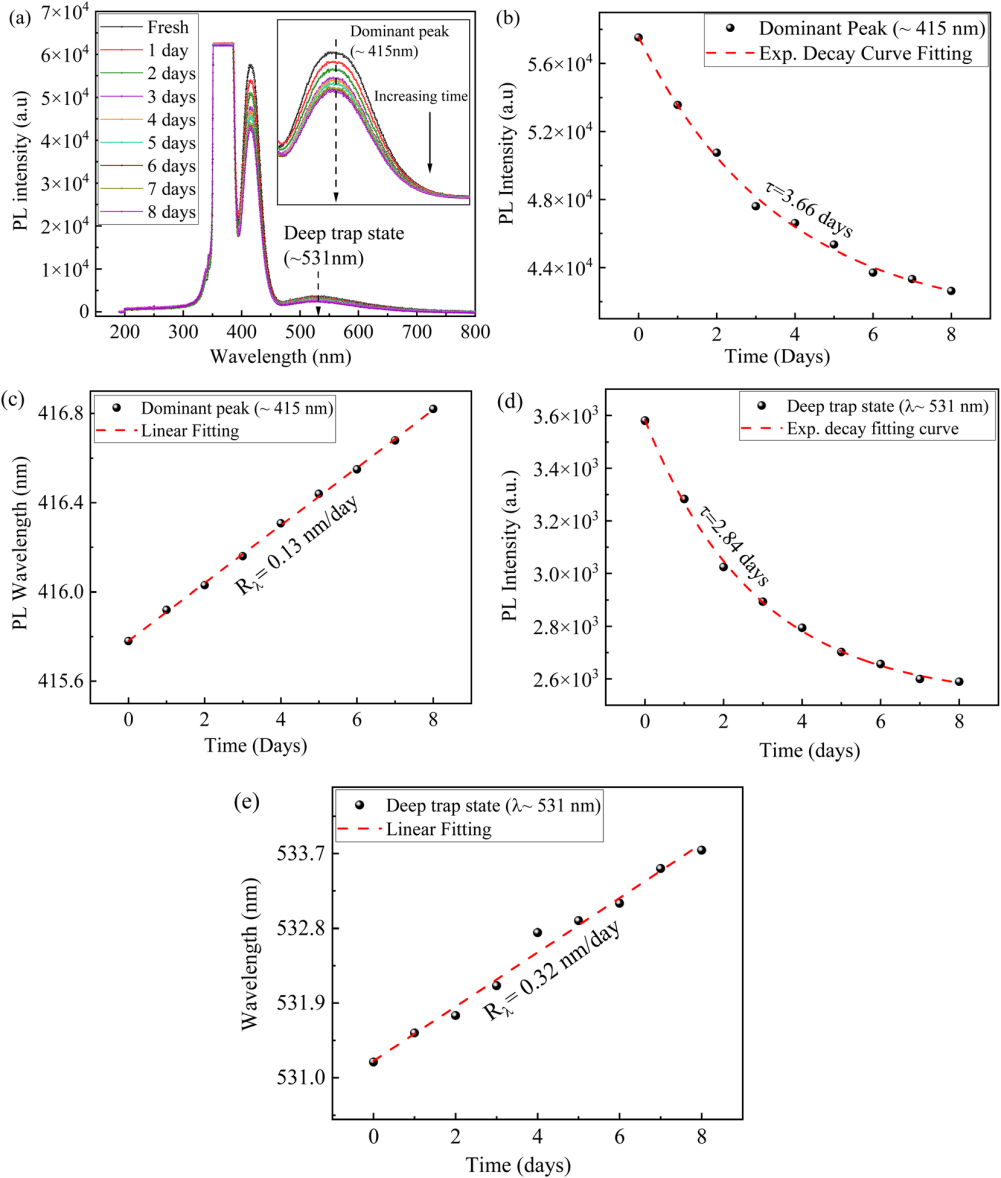
(a) Temporal evolution of PL spectrum for a CsI/NaCl film made from the aqueous solutions with 1 : 1 of CsI to NaCl, (b and c) temporal evolution of the PL peak characteristics for dominant emission (∼415 nm), and (d and e) temporal evolution of the PL peak characteristics for the deep trap state emission (∼531 nm).

Fig. 7a–e shows the time-dependent PL spectra of a CsI/NaCl film fabricated from aqueous solutions with a 1 : 1 molar ratio of CsI to NaCl and deposited at 50 °C under ambient conditions (∼50% RH), and monitored over 8 days. Beyond this, significant humidity fluctuations, especially due to rainfall, led to noticeable PL degradation and which is well consistent with the known hygroscopic nature of halide salts.^40–42^ Moreover, in most studies, long-term stability is usually assessed under controlled environments such as vacuum, inert atmospheres, or with coating/encapsulation strategies to prevent moisture- and oxygen-induced degradation.^50–53^ Thus, while extended testing under such protective conditions is typically required for device-level applications, the 8-day ambient test reported here serves as a qualitative evaluation of this CsI/NaCl (1 : 1) film's environmental stability in a realistic, and unprotected setting.

Fig. 7a shows a gradual decrease of PL intensity of dominant peak (∼425 nm) by ∼26% over this 8-day degradation period under ambient conditions (∼50% RH), indicating a transient enhancement from NaCl incorporation (Fig. 2c), followed by accelerated long-term degradation compared to pure CsI films.^13^ This faster degradation in the CsI/NaCl (1 : 1) films may be attributed to various factors such as photo-induced migration of ions or the formation of trap sites, oxidation, and diffusion of moisture in an open environment, leading to enhanced non-radiative recombination.^54–57^

Furthermore, the higher interface and grain boundary densities of the finer, mixed-phase grains (region (iii), Fig. 2a) present inside CsI/NaCl (1 : 1) films, make them more prone to moisture-induced degradation caused by water ingress and ionic migration^58–60^ and since this region (iii) (Fig. 2a) predominantly contributes to the PL emission of these films (Fig. S2), their rapid degradation under ambient conditions significantly impacts the overall optical performance of such CsI/NaCl (1 : 1) films. This interpretation aligns with prior studies on halide systems, where humidity-driven degradation was observed to initiate at grain boundaries, with smaller grain sizes accelerating such failure due to enhanced water diffusion and ionic transport across defects.^58–60^

Thereafter, the temporal evolution of the PL intensities for both dominant peak (∼415 nm) (Fig. 7b) and deep trap state peak (∼531 nm) (Fig. 7d), can be approximated *via* a similar approach employed in TCSPC decay curve analysis^61^ and based on that we assumed a exponentially decaying behaviour of PL intensity over time and is given by eqn (2):

2 *I*(*t*) = *I*_0_ + *A* e^−*t*/*τ*^

where *I*_0_ is the PL residual or baseline offset, *A* is the initial decay amplitude, and *τ* is the characteristic time for photostability degradation. Based on this curve fitting, we obtained characteristic times (*τ*) of 3.6 for dominant emission (Fig. 7b), and 2.84 days for deep trap state emission (Fig. 7d), respectively, indicating towards longer PL stability of dominant emission compared to deep trap state emission.

Fig. 7c and e reveal the linear rightward shift of both dominant and deep trap state peak. Using linear regression to fit the data in Fig. 7c and e, we obtained the values of 0.13 nm per day and 0.32 nm per day for the increase rates of the peak wavelengths (*R*_λ_) for dominant emission and deep trap state emission, respectively. The lower value of *R*_λ_ for dominant emission further validates their enhanced long-term stability in comparison to deep trap emission.

**Fig. 8:**
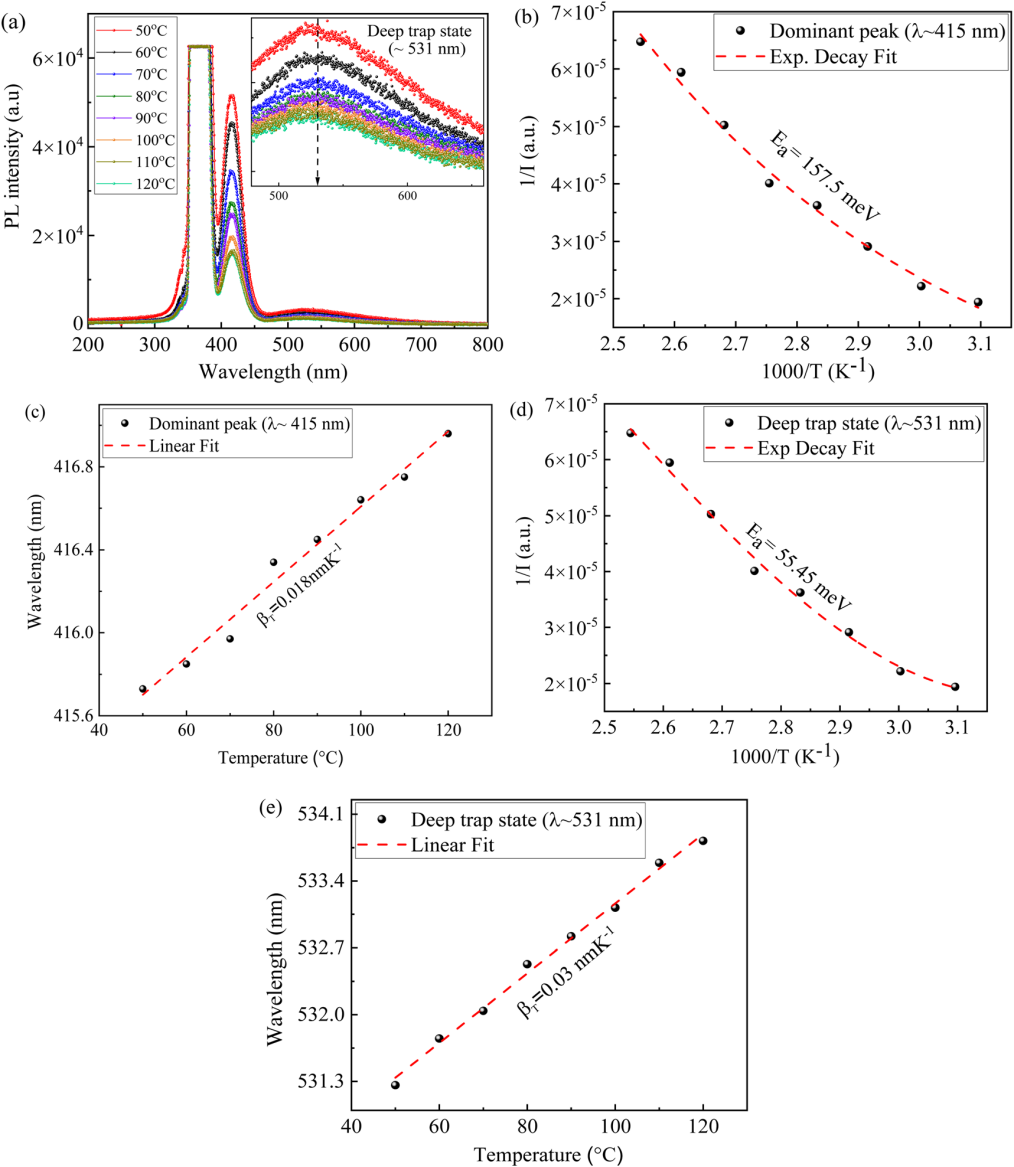
Temperature dependent Photoluminescence of CsI/NaCl (1 : 1) thin films with (a) combined spectra, (b and c) analysis for dominant emission peak (∼415 nm), (d and e) analysis for deep level emission peak (∼531 nm).

Fig. 8a presents the temperature dependent PL spectra of for a CsI/NaCl film, prepared *via* aqueous solutions with 1 : 1 molar ratio of CsI to NaCl, and it is evident that the peak intensity and peak wavelength for both the PL emission and the deep trap state emission vary with temperature, as shown in Fig. 8a–e.

From the PL spectra in Fig. 8a, we determine the variations of the PL peak intensity and wavelength as well as the peak intensity and wavelength of the deep trap state emission with temperature and present the results in Fig. 8b–e. The decrease in the PL peak intensity with increasing temperature can be ascribed to the increase of the dissociation of excitons and enhanced phonon activities at high temperatures.^62^ The temperature dependence of the emission peak intensity can be expressed as:^62^3
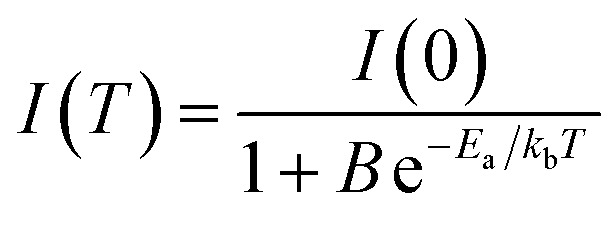
where *I*(*T*) and *I*(0) are the PL peak emission intensities at temperatures of *T* and 0 K, respectively, *B* is a constant, *k*_b_ is the Boltzmann constant, and *E*_a_ is the activation energy.

Using eqn (3), we fit the data in Fig. 8b and d and included the fitting curves in the corresponding figures. It is evident that eqn (3) describes well the temperature dependence of the emission peak intensity with activation energies of 157 meV and 55 meV for the dominant PL emission (∼415 nm) and the deep trap state emission (∼531 nm). The larger thermal activation energy for dominant PL emission further validates its higher stability in comparison to deep trap state, as initially proposed by Fig. 7b–e. Moreover, it highlighted the low energy threshold required for the recombination of charge carriers trapped in deep trap states, such as self-trapped excitons, than the dissociation of free and/or bound excitons for the PL emission. Such behaviour is commonly observed in halide materials, including CsI, due to strong exciton–phonon coupling.^63–65^

Fig. 8c and e revealed the linear increase of the peak wavelength with temperature for both dominant emission (∼415 nm) and the deep trap state emission (∼531 nm). The temperature dependence of the emission peak wavelength with a first order approximation can be expressed as:^62^

4 *λ*(*T*) = *λ*(*T*_0_) + *β*_T_Δ*T*

for |Δ*T*/*T*_0_| ≪ 1. Here, *λ*(*T*) and *λ*(*T*_0_) are the emission peak wavelengths at temperature *T* and a reference temperature *T*_0_, and *β*_T_ is a factor determining the temperature dependence of the emission peak wavelength.

Using eqn (4) to fit the data in Fig. 8c and e, we obtain the numerical values of *β*_T_ as 0.018 nm K^−1^ and 0.03 nm K^−1^ for the PL emission and deep trap state emission, respectively. The smaller numerical value of 0.018 nm K^−1^ than 0.03 nm K^−1^ further confirms greater thermal stability of dominant emission (∼415 nm) relative to deep trap state emission (∼531 nm).

## 4. Conclusions

This study demonstrates that NaCl incorporation effectively modulates the structural and opto-electronic behaviour of CsI thin films, enhancing photoluminescence and suppressing non-radiative recombination. Humidity-dependent analysis identified ∼50% RH as the optimum for a stable emission, while higher humidity levels led to performance degradation owing to moisture-induced defects and ion migration. XRD and SEM/EDS results further supported the formation of interface-rich domains and structural changes linked such effects. Additionally, distinct emission features and varying wavelength shift rates between dominant and deep trap states suggest differences in their thermal and ambient stability. Overall, this work presents a simple, cost-effective, and low temperature green pathway to achieve improved luminescent stability in halide thin films, suitable for eco-friendly optoelectronic applications under ambient conditions.

## Conflicts of interest

The authors declare no competing financial interest.

## Supplementary Material

RA-015-D5RA05682A-s001

## Data Availability

All possible experimental and analysed results have been included in this manuscript explicitly. No other new data has been generated by any further experiments/analyzation. Supplementary information is available. See DOI: https://doi.org/10.1039/d5ra05682a.
