# Supplementary material for: Effect of NaCl on the luminescent behavior of CsI thin films
Source: RSC Adv. 2025 Oct 6;15(44):36993–7005. doi: 10.1039/d5ra05682a (PMC12498220; doi:10.1039/d5ra05682a)
Supplement: RA-015-D5RA05682A-s001 [file RA-015-D5RA05682A-s001.pdf]

Supplementary Information (SI) for RSC Advances.
This journal is © The Royal Society of Chemistry 2025

Supplementary Information

Effect of NaCl on the luminescent behavior of CsI thin films

Saurabh Singh [a)] [1], Xiyu Wen [b)], Fuqian Yang [a)]

[a)] Materials Program, Department of Chemical and Materials Engineering, University of Kentucky, Lexington, KY 40506, USA

[b)] Center for Aluminium Technology, University of Kentucky, Lexington, KY 40506, USA

1. EDS Studies

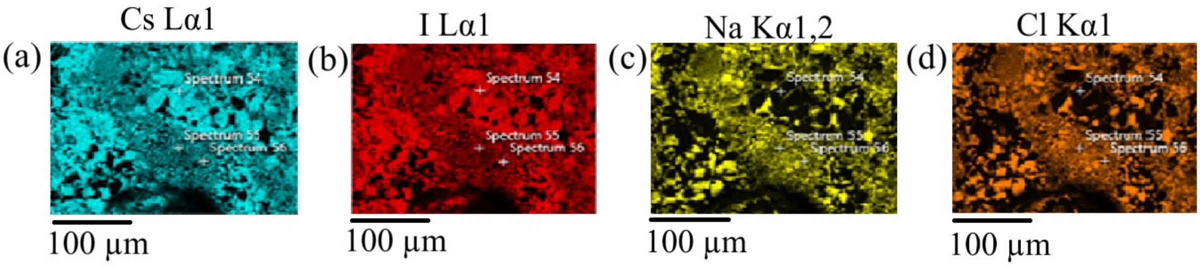

Fig. S1: EDS areal mapping for the CsI/NaCl (1:1) thin films

Table S1: Chemical composition of the (i) region.

Elements	Atomic %	Relative error
Cs	41.7	1.12
I	41	1.18
Na	12.2	0.52
Cl	4.7	0.78

Table S2: Chemical composition of the (ii) region.

Elements	Atomic %	Relative error
Cs	1.43	0.92
I	1.57	0.91
Na	41.2	0.61
Cl	41.4	0.89

Table S3: Chemical composition of the (iii) region.

Elements	Atomic %	Relative error
Cs	15.85	0.19
I	15.49	0.19
Na	22.98	0.09
Cl	19.33	0.11

[1] *Corresponding author. E-mail: ssi303@uky.edu (Saurabh Singh)*

2. PL studies

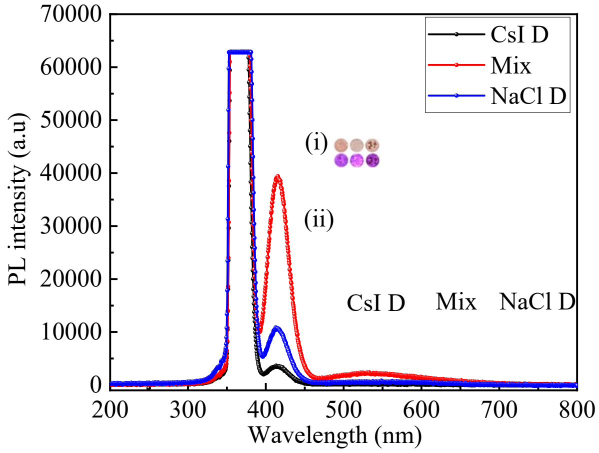

Fig. S2: PL spectra for the i (CsI dominated: CsI D), ii (NaCl dominated: NaCl D) and iii (mixed) regions. (inset: optical images of three regions under (i) room light and (ii) UV light of 365 nm in wavelength).

3. XRD Studies

3.1 Reproducibility Test for CsI/NaCl films

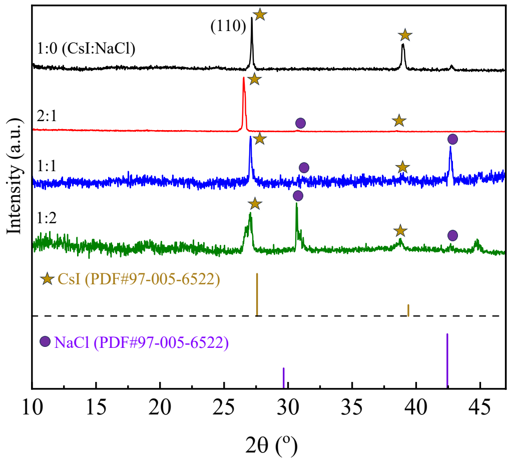

Fig. S3: XRD spectra of CsI/ NaCl films prepared via aqueous solution of CsI to NaCl in four different ratios, demonstrating the reproducibility of trend shown in Fig. 2 (b) (manuscript).

3.2 NaCl film deposited on Cu substrate

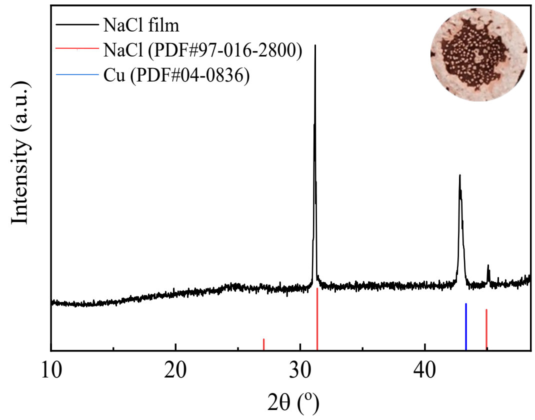

Fig. S4: XRD Spectra for NaCl film prepared via aqueous solution of NaCl and drop casted on Cu substrate (inset: Optical image of as-prepared NaCl film on Cu substrate)

4. PLQY measurements

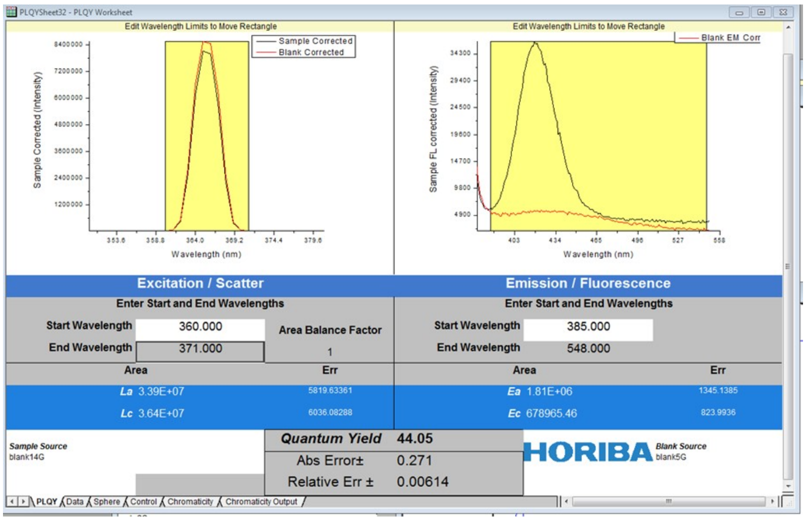

Fig. S5: Photoluminescence Quantum Yield (PLQY) measurements for CsI/NaCl (1:1) films

5. Photoluminescence (PL) intensity vs lattice strain spectra

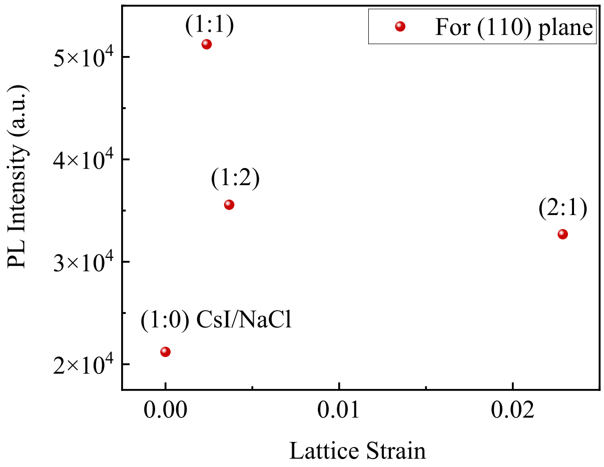

Fig. S6: Variation of PL intensity with strain induced inside CsI lattice due to introduction of different molar amounts of NaCl in CsI/NaCl films
